# Strong displayed passion and preparedness of broadcaster in live streaming e-commerce increases consumers' neural engagement

**DOI:** 10.3389/fpsyg.2022.674011

**Published:** 2022-09-13

**Authors:** Xiaoyu Yu, Yajie Li, Kexin Zhu, Wenhao Wang, Wen Wen

**Affiliations:** ^1^Laboratory Neuro-Entrepreneurship, Shanghai University, Shanghai, China; ^2^School of Management, Shanghai University, Shanghai, China

**Keywords:** live streaming, e-commerce, entrepreneurial passion, consumer engagement, inter-subject correlation, ISC, EEG

## Abstract

Live streaming shopping, the streaming of real-time videos promoting products that consumers can purchase online, has recently been a booming area of e-commerce, especially during the COVID-19 pandemic. The success of live streaming e-commerce largely relies on the extent to which the broadcaster can get consumers engaged by the live stream. Thus, it is important to discover the antecedents of consumer engagement in such a context. Drawing on consumer engagement and neuroscience literature, this study used electroencephalography inter-subject correlation (EEG-ISC) to explore how broadcasters' entrepreneurial passion during live streaming videos influenced consumers' neural engagement as they watched the live streaming videos. We used the framework of displayed passion and preparedness from the entrepreneurial passion literature to predict consumer engagement. We found significant ISC for strong displayed passion, while preparedness had partially significant effects on the first, second, and summed components of ISC. The interaction effects of these two factors on the first and summed components of ISC were partially significant. Strong displayed passion and preparedness activated the left and right prefrontal regions of the consumers' brains. These findings indicate that broadcasters' displayed passion and preparedness can influence consumer engagement in live streaming e-commerce settings. Our findings suggest that a scientific approach could be used to improve a broadcaster's performance by testing ISC during rehearsals before live streaming.

## Introduction

Streaming media technology has become popular in a range of industries, especially in e-commerce settings. E-commerce conducted *via* live stream is a trend that grew tremendously during the lockdown periods of the COVID-19 pandemic (PwC, 2020). According to a research report from KPMG (2020), the overall market size of live streaming e-commerce in China reached 433.8 billion RMB in 2019, with a 210% year-on-year growth and a penetration rate of 4.1%. Live streaming e-commerce has also opened up a market for business-to-business (B2B) e-commerce (Yu et al., 2022b). For example, a technology company used an artificial broadcaster to sell a plane in a live stream. Live stream empowers the e-commerce industry to open up a new era of online shopping.

Broadcasters are the core agents of live streaming shopping influencing consumers' internal cognitive processes such as evaluations of the trustworthiness of products (Wongkitrungrueng and Assarut, 2020). Broadcasters demonstrate the functions of products, give extremely detailed introductions to the products, answer specific questions in real-time, and organize interactive activities to entertain and attract potential consumers to “follow them” or buy products. Consumers who are cognitively engaged by live streaming shopping tend to be actively involved in real-time by watching for entertainment purposes, purchasing products, “following” the broadcasters, pressing the “like” button, and posting comments. Such interactions help the broadcaster's visibility and influence because the platform detects that many consumers are showing interest, which leads to the broadcaster being recommended to more consumers. With deep consumer engagement, broadcasters can build a strong and loyal customer base and thus improve their performance (Itani et al., 2019). Understanding how to motivate consumers to become actively engaged in a live stream is therefore important for broadcasters (Sashi, 2012; Brodie et al., 2013; Baldus et al., 2015; Kumar and Pansari, 2016; Zhang et al., 2020).

Many consumer engagement studies have considered online shopping *via* webpages (e.g., Ashraf et al., 2016), but the antecedents of consumer engagement in live streaming shopping have seldom been considered. A broadcaster's entrepreneurial passion may be a significant antecedent of consumer engagement. Entrepreneurial passion is an intense affective state accompanied by cognitive and behavioral manifestations of high personal value in entrepreneurial activities (Chen et al., 2009). Broadcasters must decide what kind of product they are going to introduce on their channel. Like entrepreneurs, they need to capture their audience's preferences precisely (Yu and Wang, 2021; Young et al., 2022). Passionate broadcasters pay attention to how to exploit opportunities (Yu et al., 2020). After deciding what products to sell, they must also prepare by deciding how they will introduce the products to consumers, including information about the product function, designing purposes, materials attribution, and so on. In addition to undertaking such preparation, passionate broadcasters often express their passion for the product during the live stream, which can attract more consumers to join the stream and stay watching (Chen et al., 2009). Thus, how entrepreneurial passion influences consumer engagement is worthy of exploration (Bowden, 2009; Ahn and Back, 2018).

In this study, we aimed to determine the effects of a broadcaster's entrepreneurial passion on the engagement of consumers watching live streaming videos by investigating the viewers' cognitive processes. To capture real-time consumer engagement and cognitive processes, we used electroencephalography inter-subject correlation analysis (EEG-ISC) and screened real live streaming shopping videos from the largest e-commerce platform in China, Taobao.com. As a report from a reliable monitoring platform (www.zhigua.cn) indicated that food has become the most popular product in live streaming shopping, we used live streaming videos selling food as our stimulus. The videos were rated on two dimensions of entrepreneurial passion—displayed passion and preparedness—by two researchers and one practitioner from a top multi-channel network (MCN) company in China and then labeled as having strong/weak displayed passion and strong/weak preparedness. The videos were then randomly assigned to participants wearing electrodes recording an EEG while they were watching. We then identified correlated components in the EEG recordings to quantify the strength of ISC across participants watching the same video. Finally, we applied analysis of variance (ANOVA) to differentiate ISC within 2 × 2 scenarios. The results revealed that for displayed passion, all of the ISC components were significantly different between the strong and weak groups. For preparedness, there was a significant difference between the groups except for the third ISC component, and the interaction of displayed passion and preparedness was significantly different between groups on the first ISC component and the summed ISC.

This study makes contributions to both theory and practice. First, our findings provide new insight into the antecedents of consumer neural engagement from a micro-foundation perspective. Second, we extend the literature on entrepreneurial passion into a new setting. Third, we apply a new method to capture consumer neural engagement at a collective level. In terms of practice, our findings could inspire broadcasters to consider entrepreneurial passion as a significant element of their performance. Our method could be used to monitor how well they perform during rehearsals. Companies could also use the method to train broadcasters and predict their performance before a formal live streaming show.

## Theoretical background

### Consumer engagement

Consumer engagement is an emerging research topic in the study of e-commerce (e.g., Patterson et al., 2006; Brodie et al., 2013; Vivek et al., 2014; Dessart, 2017). Consumer engagement in a virtual community involves specific interactive experiences between consumers and a brand, and/or other members of the community (Brodie et al., 2013). Such engagement is critical in live streaming e-commerce settings for several reasons. First, consumer engagement is not only a strategic imperative for establishing and sustaining a competitive advantage but also a valuable predictor of future business performance (Zhang et al., 2020). It can be a primary driver of sales growth (Neff, 2007) and enhance profitability (Voyles, 2007). Second, real-time interaction between consumers and broadcasters gives consumers a feeling of social presence (Gefen and Straub, 2004; Hou et al., 2021), in which consumers and the broadcaster experience synchronous communication. This helps to build trust and decrease consumers' doubts about virtual products (Wongkitrungrueng and Assarut, 2020). Third, live streaming e-commerce allows consumers to participate in a socially interactive shopping experience beyond simple purchase behavior (Brodie et al., 2013). As the interaction between consumers and broadcasters occurs in real-time and can be seen by others, consumer engagement during live streaming can not only enhance a viewer's own shopping experience but also encourage other potential consumers to interact.

Brodie et al. (2013) pointed out that consumer engagement is a context-dependent, psychological state characterized by fluctuating intensity levels that occurs within dynamic and iterative engagement processes. It is a multidimensional concept containing cognitive, emotional, and behavioral dimensions (Park and Lee, 2008). However, studies have neglected the multi-dimensional characteristics of consumer engagement (Mollen and Wilson, 2010; Hollebeek, 2011). There is a need to expand existing self-report measures of consumer engagement and use real-time measurement in live streaming settings to describe the synchronous reactions that occur during consumer engagement (Fugate, 2007). A promising approach to examining and quantifying how live streaming influences consumer engagement is the use of EEG-ISC (Cohen and Parra, 2016; Imhof et al., 2020). EEG-ISC measures the consistency and similarity of complex and naturalistic stimuli-evoked brain responses across participants who are exposed to the same message, which yields a continuous, nonverbal measure of collective engagement that is well suited to quantifying the effects of mass media messages at the neural level (Hasson, 2004; Hasson et al., 2010; Dmochowski et al., 2012; Lahnakoski et al., 2014; Imhof et al., 2017). Thus, EEG-ISC can be used to capture consumer neural engagement in live streaming settings in terms of the extent to which a participant's brain becomes engrossed in a stimulus (Shane et al., 2020). Neural engagement could reflect the focus and attention on the stimulus (Barnett and Cerf, 2017). Meanwhile, compared with other ISC-based approaches such as fMRI-ISC, EEG-ISC has advantages in its temporal resolution, which is in the order of milliseconds. Such a high temporal resolution is promising for characterizing the reception of fast-paced audiovisual messages (Imhof et al., 2020). These characteristics enhance the scalability of the method and provide the potential to integrate cognitive neuroscientific approaches into industrial pre-testing, such as during rehearsals before live streaming. Recent EEG studies in classroom settings, the cinema, and during music consumption have illustrated this potential (Barnett and Cerf, 2017; Dikker et al., 2017; Poulsen et al., 2017; Cohen et al., 2018; Madsen et al., 2019). Research has indicated that ISC can be a proxy for participant engagement arising from attention or relevance-based factors (Dmochowski et al., 2012; Hasson et al., 2012; Cohen et al., 2017; Imhof et al., 2017), suggesting that it can serve as a successful marker of engagement in live streaming videos. Thus, this study aimed to explore how live streaming influences consumer engagement from a neural perspective.

### Entrepreneurial passion

Entrepreneurial passion is an intense affective state accompanied by cognitive and behavioral manifestations of high personal value (Chen et al., 2009). As noted above, displayed passion and preparedness are two dimensions of entrepreneurial passion (Baron, 2008). Displayed passion, or appearing enthusiastic, is the affective dimension of passion (Shane et al., 2020). Preparedness, or how well prepared an individual is for a certain activity, is the cognitive dimension of entrepreneurial passion (Chen et al., 2009). Various studies have reported that entrepreneurial passion can motivate audiences to engage in certain economic activities (Smilor, 1997; Chen et al., 2009; Shane et al., 2020). For example, Shane et al. (2020) found that the passion entrepreneurs displayed during their crowdfunding pitches promoted the neural engagement and investment decisions of investors. Chen et al. (2009) found that only preparedness, but not displayed passion, increased the interest of investors in crowdfunding pitches.

Displayed passion and preparedness may also influence consumer engagement in live streaming settings. Displayed passion is often critical for convincing target audiences to invest their money and time (Chen et al., 2009). Broadcasters perform passionately, which helps them to attract consumers' attention, persuade them to make buying decisions, and create enduring and intimate relationships with them (Sashi, 2012). Thus, we hypothesize as follows:

H1: ISC has a positive relationship with displayed passion.

A well-prepared broadcast with a thoughtful plot and effective expression reveals the effort the broadcaster has invested. A well-prepared broadcaster makes the key point stand out and the content easy to understand, which attracts consumers to watch and stay on the channel. Broadcasters also prepare interactive events, such as lucky draws, which increase consumers' interest. Thus, preparedness might stimulate a high level of ISC and we hypothesize as follows:

H2: ISC has a positive relationship with preparedness.

## Materials and methods

### Participants

Forty-three participants (17 males; aged 18–29 years, *M*_age_ = 22.91, *SD* = 2.42) with normal hearing and normal or corrected-to-normal vision took part in the experiment. None of the participants had a history of neurological or psychological disorders and all were right-handed. All of them completed the pre-test and the whole EEG experiment. Data from one participant were excluded from the analysis because of technical failure during the EEG experiment. We assessed the participants' live streaming shopping experience using three questions. The first was whether they had a prior experience with live streaming shopping. If they answered “yes,” then they were asked, “Do you prefer shopping *via* live stream? (yes or no)” and “How frequently do you shop *via* live stream? (1 = not at all, 7 = very frequently).” Sixteen of the participants had experienced live streaming shopping before the experiment and three of them indicated that they preferred live streaming shopping for e-commerce (*M*_frequency_ = 3.13, *SD* = 1.54). After completing the experiment, the participants received 50 RMB or a gift of equal value as a reward. To simulate the real process of live streaming shopping, we gave each participant 500 RMB to shop with, which is much lower than the total price of all the products demonstrated in the videos. If the participant selected the most popular product, he or she received a floating payment of 30 RMB.

### Stimulus material

We collected stimulus material from Taobao.com, which is the biggest e-commerce platform in China. As noted above, we only used live streaming videos selling food as our stimulus materials. We randomly chose 50 videos from 10 to 30 September 2020. We recorded the selected live streaming videos and chose the same recording software to avoid effects from differences in brightness, sound, and optical flow influencing the experimental results. All the videos included an introduction to the products (including characteristics and price) and hawking. We recorded a minimum of 2 min and a maximum of 3 min of the live stream to ensure that typical content was captured.

Next, two researchers with expertise in entrepreneurial passion and one practitioner, the manager of an MCN company in the live streaming e-commerce industry, were asked to independently rate the broadcasters' displayed passion and preparedness using a scale from Chen et al. (2009), which has been widely used to study entrepreneurial passion (e.g., Davis et al., 2017; Shane et al., 2020; Alison et al., 2022). The statements about displayed passion were as follows: “When introducing the product, the broadcaster's body language is very rich,” “When introducing the product, the broadcaster's facial expression is very rich,” and “When introducing the product, the broadcaster's eyes are glowing,” and “When introducing the product, the broadcaster has a very high tone and intonation.” The statements about preparedness were as follows: “The video content had substance,” “The video was thoughtful and in-depth,” “The video was coherent and logical,” “The broadcaster articulated the relationship between the product and its function,” and “The broadcaster cited facts to support his/her arguments.”

The mean score for displayed passion was 4.35, with a standard deviation of 1.10. The mean score for preparedness was 4.58, with a standard deviation of 0.95. An inter-rater reliability assessment was conducted to confirm the level of agreement across the three different evaluators. Krippendorff's α was 0.827, which exceeded the “very good” threshold value of 0.80 (Landis and Koch, 1977). We categorized the videos that scored one deviation above the average as strong and those that scored one deviation below the average as weak, based on the three evaluators' scores. Videos that did not score in this range were deleted because it was hard to tell the difference between high passion and low passion. Seven videos were excluded through this process. Finally, 15 videos were rated as strong displayed passion and strong for preparedness, seven were rated as strong for displayed passion and weak for preparedness, seven were rated as weak for displayed passion and strong for preparedness, and 16 were rated as weak for displayed passion and weak for preparedness. *T*-tests for these four categories showed that there were significant differences among groups of videos in the broadcaster's displayed passion (*M*_strong_ = 4.75, *SD* = 0.41; *M*_weak_ = 3.88, *SD* = 0.35, *t* = −7.4438, *p* < 0.001) and preparedness (*M*_strong_ = 4.36, *SD* = 0.40; *M*_weak_ = 3.33, *SD* = 0.42, *t* = −8.2437, *p* < 0.001). To confirm that our categorization was valid, we also asked the participants to rate the broadcaster's displayed passion and preparedness using the same scale after watching each video. We assessed the agreement in the scores between the evaluators and the participants by comparing the average of the three evaluators' scores with the average of the participants' scores. Krippendorff's α was 0.662, which is higher than the “good” threshold value of 0.60 (Landis and Koch, 1977). The *t*-tests of the participants' scores for the four categories confirmed the differences in displayed passion (*M*_strong_ = 4.75, *SD* = 0.41; *M*_weak_ = 3.88, *SD* = 0.35, *t* = −7.4438, *p* = 0.0000) and preparedness (*M*_strong_ = 4.36, *SD* = 0.40; *M*_weak_ = 3.33, *SD* = 0.42, *t* = −8.2437, *p* = 0.0000) that had been indicated by the raters' scores.

### Procedure

Before the experiment (*t*1), we obtained permission for our study from the Technology Ethics Committee of Shanghai University. We then posted a recruitment poster stating the experiment's purpose, location, duration, requirements, remuneration, and contact information. Before the experiment, the participants were asked to clean their scalp with shampoo. They were also asked to sign an informed consent form confirming that they understood that the EEG procedure was safe and would be kept confidential. They were then asked to complete a pre-test questionnaire covering general demographic information (name, gender, age, education level, and handedness) and their spending behaviors (level of spend per month, live streaming shopping experience). Next, the experimenter led the participants into a professional EEG lab with sound, light, and magnetic insulation one by one. Each participant was fitted with a 64-channel electrode cap, external electrodes, and conductive paste. This usually took 20 min.

**Figure 1:**
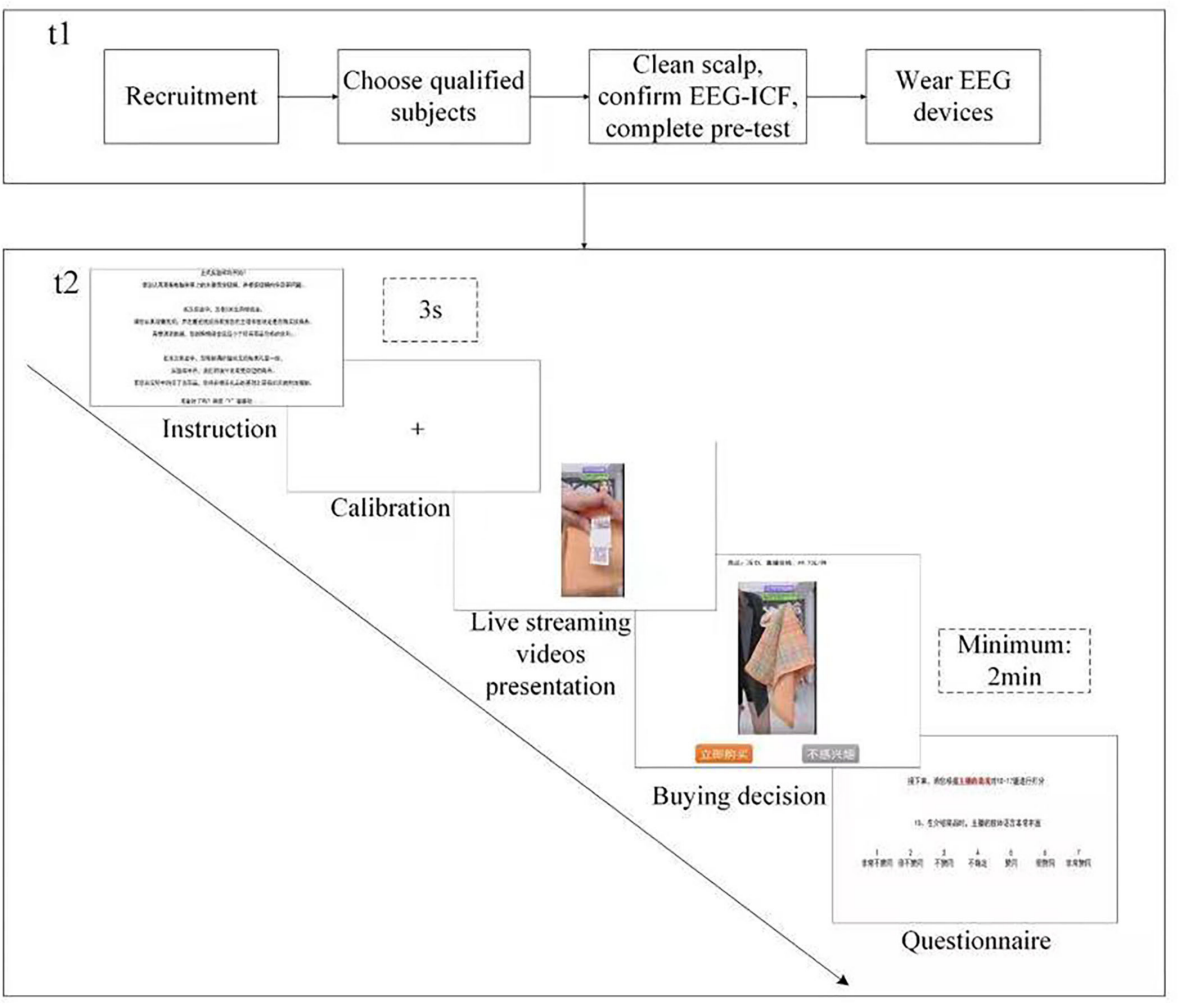
Experimental procedure.

During the experiment (*t*2), each participant was randomly shown 15 of the 45 live streaming shopping videos. Each video was viewed by at least 12 participants. The videos were presented *via* E-prime 2.0 at a resolution of 1,920 × 2,080 pixels on a 24" flat screen monitor, located around 55–75 cm in front of the participant. First, the instructions for the experiment were presented on the screen for at least 30 s. When the participant pressed the space key, the next process was a familiarization exercise. In the formal round, a 3-s fixed-image was shown before each video. After a 2-min video clip, the participants had to make a purchase decision using the “Buy Now” and “Not Interested” buttons displayed on the screen to proceed to the rest of the experiment. At the end of each video, a questionnaire about the displayed passion and preparedness of the broadcaster (Chen et al., 2009) was presented on the screen and the participants were asked to score that broadcaster between one (not at all passionate) and seven (very passionate). Each experiment lasted for approximately 1.5 h, with a break of 5–10 min after the eighth video. The experimental procedure is shown in Figure 1.

### EEG acquisition and preprocessing

The acquisition of the EEG was made using an ActiCHamp amplifier with 64 channels developed by Brain Products. The EEG was sampled at 256 Hz and the electrodes were distributed on the participants' scalps following the international standard 10–20 system. The vertex electrode (Cz) was the reference electrode. To ensure data quality, scalp impedance must remain below 10 kΩ. All the participants' scalp impedance values were below 10 kΩ at the beginning of the experiment, and we also tried to adjust each electrode during the rest time between the two rounds. However, the scalp impedance of several participants was higher than 10 kΩ for several videos. We documented these instances and excluded eight EEG records from the ISC analysis. We ensured each video had at least 12 observations, which was necessary for calculating EEG-ISC according to prior research (Cohen and Parra, 2016).

We preprocessed the raw data following the procedures of prior studies (Dmochowski et al., 2012, 2014; Cohen and Parra, 2016; Cohen et al., 2017; Imhof et al., 2020). The preprocessing was done using BrainVision Analyzer software (Brain Products, 2013). Specifically, EEG data were set at 0.5 Hz for high pass and 50 Hz for notch filter. As participants tend to blink during experiments, which can affect the prefrontal region, it is necessary to eliminate the effect of blinking on the results by removing the interference signals. We removed eyeblinks using the ICA-based ocular artifact rejection function of the BrainVision Analyzer software. We manually confirmed the ocular components after the system selected the eligible components by ICA. As we had marked the live streaming videos using the E-prime program, we then segmented the EEG data according to the marks. Finally, we put the segmented EEG data into Matlab to identify the outlier samples in each specific channel. Outlier samples were those values that were four times larger than the distance between 25 and 75%, and we used zero values to replace 40 ms before and after the outliers (Cohen and Parra, 2016).

### EEG-ISC analysis

We used the open-source code for EEG-ISC developed by Parra and other scholars (available at http://www.parralab.org/isc/). EEG-ISC calculates the maximally correlated components by capturing a linear combination of electrodes that are consistent across participants and maximally correlated between them (Cohen and Parra, 2016). Compared with fMRI-ISC, EEG-ISC can detect temporal-scale response patterns to stimuli (Dmochowski et al., 2012; Cohen and Parra, 2016; Imhof et al., 2020). We used within- and between-subject covariance matrices that were averaged across all videos to calculate the correlated components. We further extracted three components to capture most of the ISC, and the corresponding correlation values were computed separately for each component and each video. We also calculated the sum of the three largest correlated components to enable comparison with previous research (Cohen and Parra, 2016). As components are temporally uncorrelated with other components and capture different sources of neural activity, we used the “forward model,” which represents the covariance between the activity of each component and the activity of each electrode location (Parra et al., 2005) to visualize the spatial distribution of different sources (Parra et al., 2005).

### Statistical analysis

We performed three major analyses. First, we used the Kolmogorov–Smirnov test and Levene's test to check whether our data satisfied the assumptions of normality and uniformity of variance required for ANOVA. Second, we applied ANOVA to test the statistical differences among different categories. Third, we applied the least significant difference (LSD) *post-hoc* analysis to provide a robust test of the differences' direction and magnitude. All analyses were performed using SPSS 20.0 for Windows.

## Results

The purpose of this study is to investigate the influence of broadcaster's displayed passion (2 levels, strong and weak) and preparedness (2 levels, strong and weak) in live streaming on consumer's neural engagement. For each component, the effect of displayed passion and preparedness was assessed *via* ANOVA. In order to examine the degree of neural engagement within participants, we measured 43 participants' EEG activity during video presentation to assess neural processing. To calculate ISC, we extracted the three most correlated components and summed the ISC of the EEG data (Cohen and Parra, 2016). We expected that strong displayed passion, strong preparedness, and the interaction between them would be associated with higher ISC than weak displayed passion and poor preparedness. As shown in **Figure 3**, the LSD *post-hoc* analysis revealed that for component 1 and the summed components, the weak displayed passion^*^strong preparedness condition was associated with significantly higher ISC than the weak displayed passion^*^weak preparedness condition (C_1_: *p* = 0.003; C_sum_: *p* = 0.001), strong displayed passion^*^weak preparedness condition (C_1_: *p* = 0.015; C_sum_: *p* = 0.000), and strong displayed passion^*^strong preparedness condition (C_1_: *p* = 0.001; C_sum_: *p* = 0.000).

### Pre-requisite for ANOVA

The Kolmogorov-Smirnov normality test was performed to confirm that the data were sampled from components with a normal distribution. The *p*-values of the Kolmogorov-Smirnov test were all larger than 0.05 (C_1_: *p* = 0.841; C_2_: *p* = 0.989; C_3_: *p* = 0.727; C_sum_: *p* = 0.336), indicating that all ISC components and summed components followed a normal distribution.

Levene's test evaluates the null hypothesis that the component variances are homogeneous. When the *p*-value is < 0.05, the null hypothesis is rejected, and it can be concluded that there is a difference between the variances of components. The results of the Levene test showed that the assumption of homogeneity of variances for the components was supported (C_1_: *p* = 0.637; C_2_: *p* = 0.530; C_3_: *p* = 0.051; C_sum_: *p* = 0.139). As the data were confirmed to follow a normal distribution and to have homogeneous variance, the pre-requisites for ANOVA were satisfied.

### ANOVA

**Figure 2:**
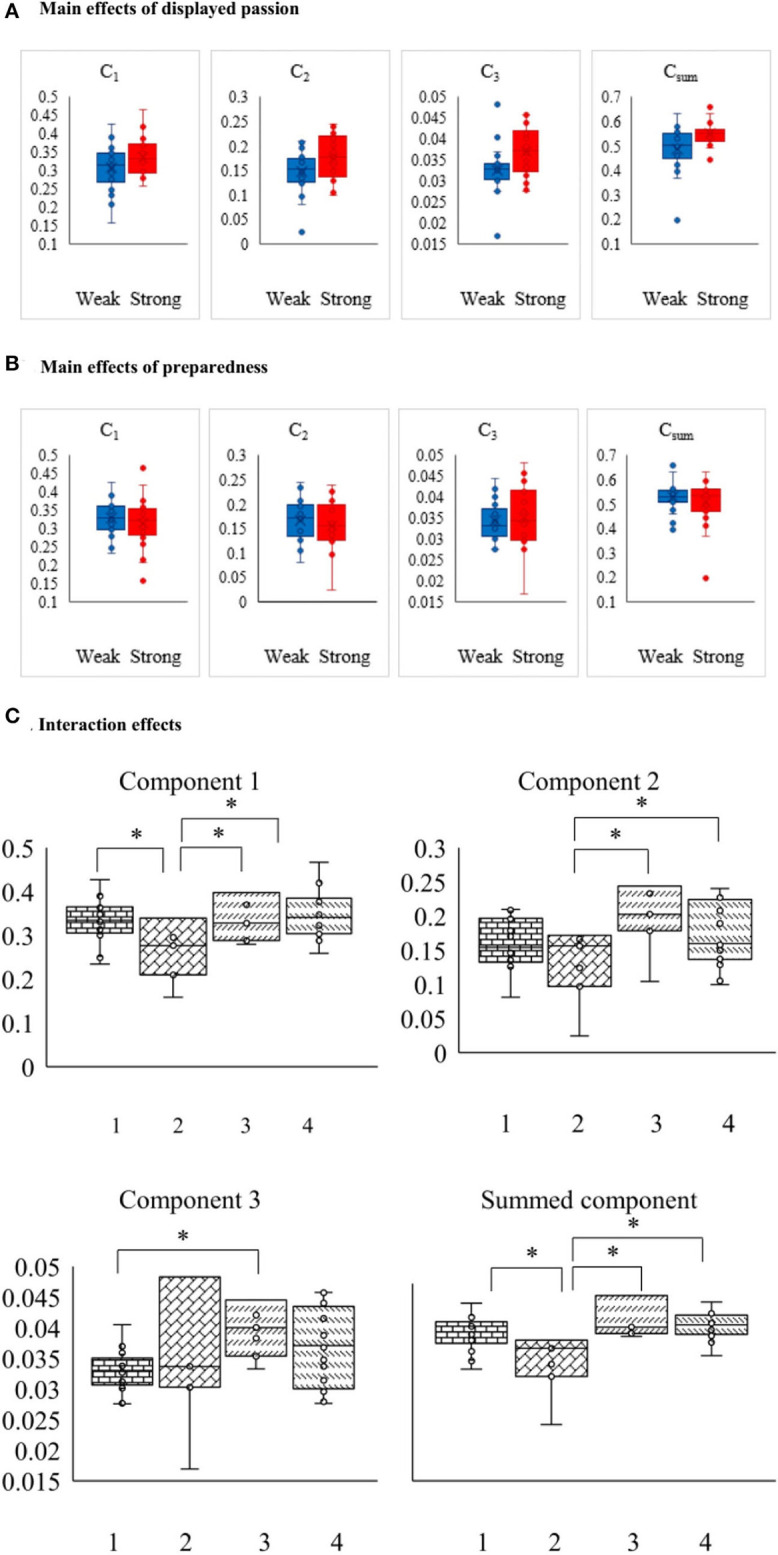
EEG-ISC detects differences during the viewing of different videos. (1) Box plots a and b show average EEG-ISC for each component, separated by video categories high (red) and low (blue) passion. We use a smaller scale unit on the third component to show differences among different videos because the third one captured least ISC among all those components. (2) Box plots c show average EEG-ISC for each component, separated by video categories (1–4). Video category 1–4 represents “weak displayed passion^*^weak preparedness,” “weak displayed passion^*^strong preparedness,” “strong displayed passion^*^weak preparedness,” “strong displayed passion strong preparedness.” We use a smaller scale unit on the third component to show differences among different videos because the third one captured least ISC among all those components.

The results of ANOVA showed that all the ISC components while viewing live streaming videos were significantly correlated with the broadcaster's displayed passion (C_1_: *F* = 6.42, *p* = 0.0154; C_2_: *F* = 6.82, *p* = 0.0127; C_3_: *F* = 6.41, *p* = 0.0155; C_sum_: *F* = 17.15, *p* = 0.0002; for details, please see Table 1). The results also confirmed that preparedness was significantly correlated with C_1_, C_2_ and C_sum_ (C_1_: *F* = 3.13, *p* = 0.0848; C_2_: *F* = 3.53, *p* = 0.0678; C_3_: *F* = 0.48, *p* = 0.4911; C_sum_: *F* = 8.01, *p* = 0.0073). As shown in Figure 2, it was possible to discern differences between the effects of different video categories on all ISC components and summed ISC. These results support our hypotheses that strong passion and preparedness in live streaming videos prompt ISC among participants.

**Table 1:** Results of two-way ANOVA comparing brain coupling during different dimensions.

	**Effect**	***F***	***p*-value**	**Sum of Squares**	**η^2^**
Component 1	Displayed passion	6.42	0.0154	0.01791	0.12
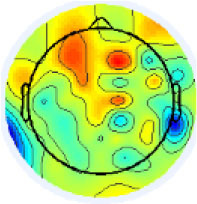	Preparedness	3.13	0.0848	0.00872	0.06
	Displayed passion × Preparedness	7.19	0.0107	0.02006	0.14
Component 2	Displayed passion	6.82	0.0127	0.01330	0.15
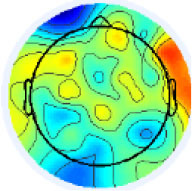	Preparedness	3.53	0.0678	0.00688	0.08
	Displayed passion × Preparedness	0.22	0.6421	0.00043	0.00
Component 3	Displayed passion	6.41	0.0155	0.00022	0.14
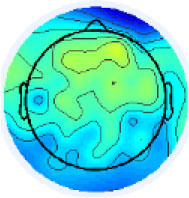	Preparedness	0.48	0.4911	0.00002	0.01
	Displayed passion × Preparedness	0.36	0.5507	0.00001	0.01
Summed component	Displayed passion	17.15	0.0002	0.06973	0.27
	Preparedness	8.01	0.0073	0.03256	0.13
	Displayed passion × Preparedness	6.20	0.0172	0.02521	0.10

We also applied a two-way ANOVA to identify the interaction effect of the two dimensions. The results revealed that the interaction of “displayed passion^*^ preparedness” reached significance for C_1_ and C_sum_ (C_1_: *F* = 7.19, *p* = 0.0107; C_sum_: *F* = 6.20, *p* = 0.0172), but not for C_2_ or C_3_ (C_2_: *F* = 0.22, *p* = 0.6421; C_3_: *F* = 0.36, *p* = 0.5507). To clarify the direction of interaction effects, we conducted a simple effects test. The results showed that with weak displayed passion the simple effects of preparedness were significant for C_1_ (*F* = 8.79, *p* = 0.031) and C_sum_ (*F* = 8.84, *p* = 0.031), but they were not significant when displayed passion was strong. With strong preparedness, the simple effects of displayed passion were significant for C_1_ (*F* = 12.07, *p* = 0.018) and C_sum_ (*F* = 12.91, *p* = 0.016). A box plot of the main effects and interaction effects is shown in Figure 2.

### *Post-hoc* analysis

We used category 1 to category 4 to represent the “weak displayed passion^*^weak preparedness,” “weak displayed passion^*^strong preparedness,” “strong displayed passion^*^weak preparedness,” and “strong displayed passion^*^strong preparedness” conditions, respectively. The results of the LSD *post-hoc* analysis are shown in Table 2.

**Table 2:** Results of LSD *post-hoc* analysis.

	**Groups**		***p*-value**	**Mean difference**	**Std. Error**
Component 1	1	2	0.003	0.0801^*^	0.025
		3	0.917	0.003	0.025
		4	0.473	−0.014	0.019
	2	3	0.015	−0.0771^*^	0.030
		4	0.001	−0.0941^*^	0.026
	3	4	0.524	−0.016	0.026
Component 2	1	2	0.104	0.035	0.021
		3	0.136	−0.032	0.021
		4	0.492	−0.011	0.016
	2	3	0.012	−0.0671^*^	0.025
		4	0.036	−0.0461^*^	0.021
	3	4	0.492	0.011	0.016
Component 3	1	2	0.947	0.000	0.003
		3	0.032	−0.0061^*^	0.003
		4	0.090	−0.004	0.002
	2	3	0.065	−0.006	0.003
		4	0.182	−0.003	0.003
	3	4	0.367	0.003	0.003
Summed	1	2	0.001	0.1151^*^	0.031
component		3	0.248	−0.036	0.031
		4	0.222	−0.028	0.023
	2	3	0.000	−0.1511^*^	0.037
		4	0.000	−0.1441^*^	0.031
	3	4	0.812	0.007	0.031

* p < 0.05.

For component 1 and the summed components, the *post-hoc* analysis showed that the ISC of category 2 was significantly higher than the ISC of the other categories (*p* < 0.05). The ISCs of categories 1, 3, and 4 were not statistically different from each other. These results suggest that for component 1 and the summed components, the “weak displayed passion^*^strong preparedness” condition was most effective in activating higher ISC.

For component 2, the analysis showed that the ISC of category 2 was significantly higher than that of category 3 (*p* < 0.05) and category 4 (*p* < 0.05), and the ISCs of categories 1, 3, and 4 were not statistically different from each other. Thus, we can conclude that the “weak displayed passion^*^strong preparedness” condition activated higher ISC than the “strong displayed passion^*^weak preparedness” and “strong displayed passion^*^strong preparedness” conditions did.

For component 3, the analysis showed that the ISC of category 3 was only significantly higher than that of category 1 (*p* < 0.05). The ISCs of categories 1, 2, and 4 were also not statistically different from each other. Thus, we can conclude that the “strong displayed passion^*^weak preparedness” condition activated higher ISC than the “weak displayed passion^*^weak preparedness” condition did.

### Sensor contribution analysis

**Figure 3:**
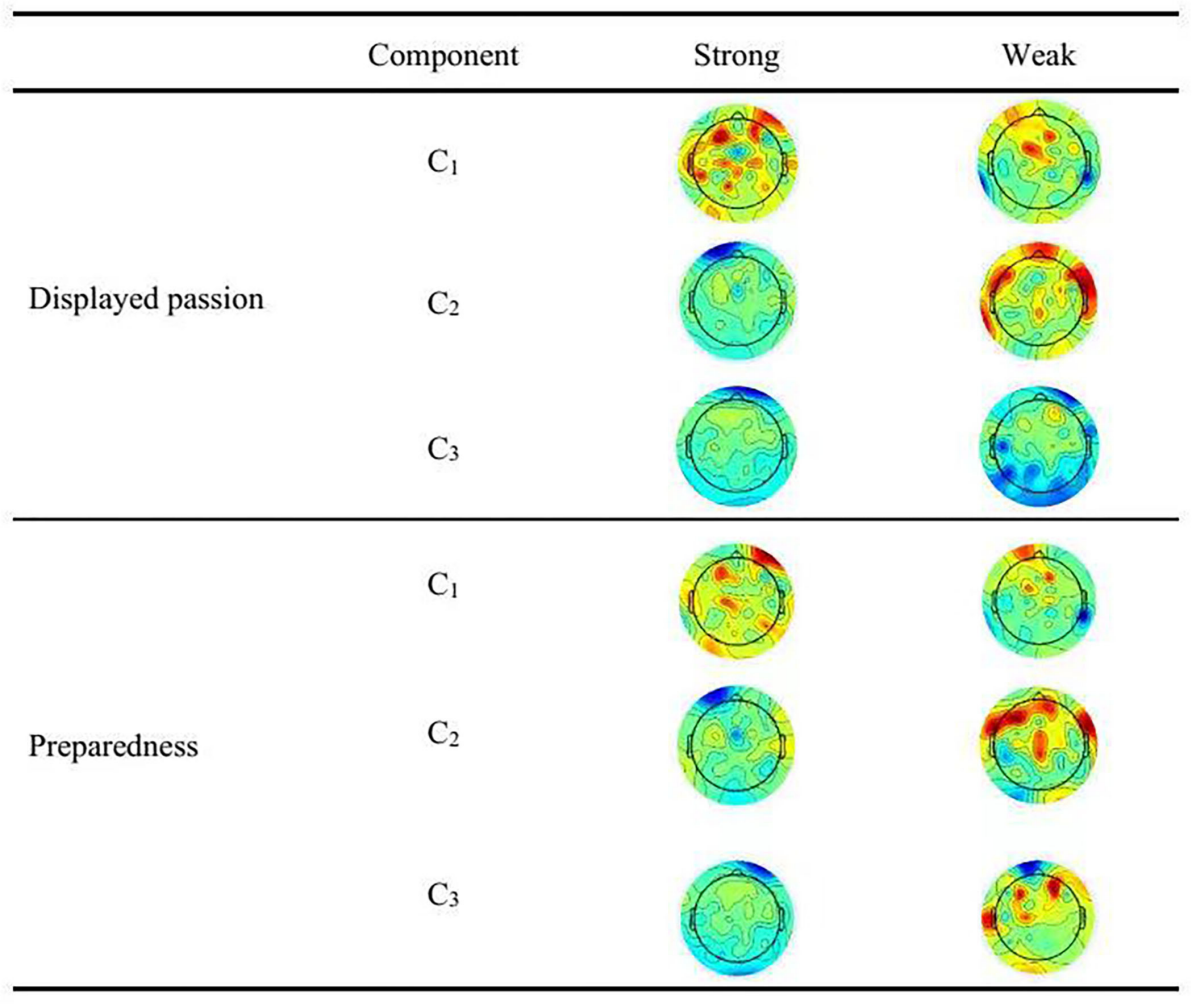
Sensor contribution. Topographical map visualizes the strength that each sensor contributes to the correlated component. Each column represents the forward model (con-elation between surface electrodes and component activity) obtained using either all stimuli together (combining responses across all participants). The maps reveal the contribution by showing interpolated magnitudes of the scalp projections, that is the forward models of the maximally correlated components C_1_ to C_3_.

Figure 3 visualizes which sensors contributed to the correlated components and reveals distinct topographies for each component using their forward projections. The spatial distribution of the components represents the covariance between a component's activity and the activity at each sensor (Dmochowski et al., 2012; Cohen and Parra, 2016). Combining the results of the ANOVA (Table 1) and sensor contribution (Figure 2) enabled us to conduct further analysis to better understand the different sources of neural engagement in the different categories, although EEG does not reflect brain regions as precisely as fMRI does.

As shown in Table 1, the activated brain region of C_2_ was the prefrontal region. It has been found that the prefrontal region mainly has functions of affective engagement and judgment (Dulabh et al., 2017). As shown in Figure 2, we found that the prefrontal region was more strongly activated by the videos with strong displayed passion than by those with weak displayed passion. This was also the case for strong and poor preparedness. Thus, the sensor contribution was in accordance with our ANOVA results. These results show that strong displayed passion motivated more neural engagement than weak displayed passion did, and the same was true for strong vs. poor preparedness.

The activated brain regions of C_2_ were the left prefrontal lobe, left frontal lobe, and right frontal lobe. The left prefrontal lobe is mainly associated with enhancing the memory of stimuli (Gabrieli et al., 1998), and the left and right frontal lobes are mainly associated with motor events (Decety, 1996). The strong displayed passion and strong preparedness videos activated the left prefrontal lobe more extensively and significantly than their weak counterparts did. This brain region is responsible for enhancing the memory of stimulus, which is related to engagement in the context of live streaming shopping. Although the weak displayed passion videos and poor preparedness videos activated the left and right frontal lobes, these areas are responsible for motor events, which are irrelevant to our setting. Thus, the sensor contribution of C_2_ was in accordance with the ANOVA results, indicating that strong displayed passion motivated more neural engagement than weak displayed passion did and strong preparedness motivated more neural engagement than poor preparedness did.

The activated brain regions of C_3_ were around the right prefrontal lobe and right frontal lobe. It has been found that the right prefrontal lobe is mainly associated with episodic memory retrieval (Buckner et al., 1996) and the right frontal lobe is mainly associated with self-awareness (Stuss, 1991). Compared with weak displayed passion, strong displayed passion activated the right prefrontal lobe extensively and significantly. This brain region is responsible for episodic memory retrieval, which is related to engagement during the live stream. Although weak displayed passion activated the right frontal lobe, this area of the brain is mainly responsible for self-awareness and is less related to engagement in the context of live streaming shopping. Therefore, the sensor contribution of C_3_ was also in accordance with the ANOVA results, indicating that strong displayed passion motivated more neural engagement than weak displayed passion did.

## Discussion

This study explored the antecedents of consumer neural engagement in a live streaming e-commerce setting, breaking new ground by studying the inter-subject neural activity of consumers in a live streaming shopping setting (Dulabh et al., 2017). We found significant EEG-ISC for videos characterized by strong displayed passion, while the effects of preparedness were partially significant for the first, second, and summed components of EEG-ISC. The conjoint effects of these two factors were partially significant for the first and summed components of EEG-ISC. Strong displayed passion was found to trigger activity in the left and right prefrontal regions of the brain, indicating that broadcasters with strong displayed passion can motivate consumers to engage with live streaming shopping. Strong preparedness triggered activity in the left prefrontal region, suggesting that well-prepared broadcasters can also motivate consumers to engage with live streaming shopping. Overall, we conclude that both strong displayed passion and strong preparedness can promote consumer engagement in live streaming shopping. Overall, we found that displayed passion was the main antecedent of consumer engagement in live streaming e-commerce; preparedness only partially enhanced consumer engagement. These two characteristics jointly enhanced consumer neural engagement.

### Theoretical contribution

Our results make three major contributions to the e-commerce literature, the entrepreneurial passion literature, and the neuroscience of EEG-ISC literature. First, we extend the micro-foundation of consumer engagement literature by reporting the antecedents of consumer engagement with live streaming e-commerce (Yu et al., 2022a). Consumer engagement is an extremely important performance indicator for live streaming e-commerce, but it is hard to find evidence of the factors influencing consumer engagement in this setting (Wongkitrungrueng and Assarut, 2020). This study found that consumers were more engaged if broadcasters displayed more passion. A well-prepared introduction to the products being sold also directly enhances consumer engagement.

Second, we contribute to the entrepreneurial passion literature by depicting the role of passion in a live streaming setting. Research has indicated the significant role of passion in a range of settings. Chen et al. (2009) revealed that preparedness but not displayed passion influenced investment decisions in a roadshow setting. On the other hand, Shane et al. (2020) found that displayed passion affected the neural engagement of investors in entrepreneurship. Our findings fill the gap among these conflicting reports by considering both cognitive passion and emotional passion in a live streaming setting. Our results indicate that in a live streaming setting, both displayed passion and preparedness are important. The role of entrepreneurial passion is different in different settings.

Third, this study is the first to apply an electroencephalography inter-subject correlation (EEG-ISC) approach to analyze consumer engagement in a live streaming shopping context. It extends existing measurements of consumer engagement to the neural level. A majority of e-commerce studies are survey-based or use secondary data, for which participants were asked to report how they felt they had been engaged (e.g., Brodie et al., 2013) or directly observable behavior (e.g., Vivek et al., 2014). However, consumer engagement is a multi-dimensional concept that is difficult to represent with a single variable due to the richness of reflections on different platforms (Cheung et al., 2014). As cognitive engagement is a fundamental mechanism of behavioral engagement (Park and Lee, 2008), applying EEG-ISC to the study of live streaming e-commerce enhances the understanding of consumer engagement by objectively quantifying cognitive engagement in this context.

### Practical contribution

This paper can inspire both broadcasters and MCN companies that nurture broadcasters. Our findings indicate the importance of entrepreneurial passion in motivating consumer engagement. Broadcasters should display a high level of passion during their live stream by using rich body language and facial expressions. They should also be diligent in their preparation before the live stream starts. For MCN companies, EEG-ISC provides a method of predicting consumer engagement before a formal live stream by detecting consumer neural engagement during a rehearsal, rather than monitoring consumer engagement behavior during the live stream. By comparing several rounds of different levels of displayed passion and preparedness, broadcasters could discover the most effective way to perform in live streaming videos.

### Limitations and future directions

This study focused on live streaming e-commerce and our findings are limited to this context, although our findings complement the results of other EEG-ISC studies. Whether passion has a universal influence in other e-commerce scenarios remains unknown. Furthermore, to avoid the effects of individual preference, we only used videos about food products as our stimulus materials. Whether the attributes of products influence consumer engagement also remains unknown and requires further study.

## Data availability statement

The raw data supporting the conclusions of this article will be made available by the authors, without undue reservation.

## Ethics statement

The studies involving human participants were reviewed and approved by Technological Ethics Committee of Shanghai University. The patients/participants provided their written informed consent to participate in this study.

## Author contributions

XY, YL, and WWe conceived and designed the experiments. WWe, YL, and WWa performed the experiments. KZ, WWe, and YL analyzed the data. YL, KZ, and XY wrote and refined the article. All authors contributed to the article and approved the submitted version.

## Funding

This research was funded by the Natural Science Foundation of China (71972126 and 71772117), the National Key Research and Development Program (2020YFB1708200), and the Innovation Program of Shanghai Municipal Education Commission (2019-01-07-00-09-E00078).

## Conflict of interest

The authors declare that the research was conducted in the absence of any commercial or financial relationships that could be construed as a potential conflict of interest.

## Publisher's note

All claims expressed in this article are solely those of the authors and do not necessarily represent those of their affiliated organizations, or those of the publisher, the editors and the reviewers. Any product that may be evaluated in this article, or claim that may be made by its manufacturer, is not guaranteed or endorsed by the publisher.

## References

[B1] Ahn J. Back K. J. (2018). Antecedents and consequences of customer brand engagement in integrated resorts. Int. J. Hosp. Manag. 75, 144–152. 10.1016/j.ijhm.2018.05.020

[B2] Alison T. H. Warnick B. J. Davis B. C. Cardon M. S. (2022). Can you hear me now? Engendering passion and preparedness perceptions with vocal expressions in crowdfunding pitches. J. Bus. Ventur. 37, 106193. 10.1016/j.jbusvent.2022.106193

[B3] Ashraf A. R. Razzaque M. A. Thongpapanl N. (2016). The role of customer regulatory orientation and fit in online shopping across cultural contexts. J. Bus. Res. 69, 6040–6047. 10.1016/j.jbusres.2016.05.019

[B4] Baldus B. J. Voorhees C. Calantone R. (2015). Online brand community engagement: scale development and validation. J. Bus. Res. 68, 978–985. 10.1016/j.jbusres.2014.09.03525965862

[B5] Barnett S. B. Cerf M. (2017). A ticket for your thoughts: method for predicting movie trailer recall and sales using neural similarity of moviegoers. J. Consum. Res. 44, 160–181. 10.1093/jcr/ucw083

[B6] Baron R. A. (2008). The role of affect in the entrepreneurial process. Acad. Manag. Rev. 33, 328–340. 10.5465/amr.2008.31193166

[B7] Bowden J. (2009). Customer engagement: a framework for assessing customer-brand relationships: the case of the restaurant industry. J. Hosp. Mark. Manag. 18, 574–596. 10.1080/19368620903024983

[B8] Brain Products (2013). Ocular Correction ICA, Vol. 49. Berlin: Brain ProductsPress Release.

[B9] Brodie R. J. Ilic A. Juric B. Hollebeek L. (2013). Consumer engagement in a virtual brand community: an exploratory analysis. J. Bus. Res. 66, 105–114. 10.1016/j.jbusres.2011.07.029

[B10] Buckner R. L. Raichle M. E. Miezin F. M. Petersen S. E. (1996). Functional anatomic studies of memory retrieval for auditory words and visual pictures. J. Neurosci. 16, 6219–6235. 10.1523/JNEUROSCI.16-19-06219.1996 8815903 PMC6579164

[B11] Chen X. P. Yao X. Kotha S. (2009). Entrepreneur passion and preparedness in business plan presentations: a persuasion analysis of venture capitalists' funding decisions. Acad. Manag. J. 52, 199–214. 10.5465/amj.2009.36462018

[B12] Cheung C. M. K. Xiao B. S. Liu I. L. B. (2014). Do actions speak louder than voices? The signaling role of social information cues in influencing consumer purchase decisions. Dec. Support Syst. 65, 50–58. 10.1016/j.dss.2014.05.002

[B13] Cohen S. S. Henin S. Parra L. C. (2017). Engaging narratives evoke similar neural activity and lead to similar time perception. Sci. Rep. 7, 4578. 10.1038/s41598-017-04402-4 PMC5496904 28676688

[B14] Cohen S. S. Madsen J. Touchan G. Robles D. Lima S. F. A. Henin S.. (2018). Neural engagement with online educational videos predicts learning performance for individual students. Neurobiol. Learn. Mem. 155, 60–64. 10.1016/j.nlm.2018.06.011 29953947

[B15] Cohen S. S. Parra L. C. (2016). Memorable audiovisual narratives synchronize sensory and supramodal neural responses. Eneuro 3, ENEURO.0203-16.2016. 10.1523/ENEURO.0203-16.2016 PMC5103161 27844062

[B16] Davis B. C. Hmieleski K. M. Webb J. W. Coombs J. E. (2017). Funders' positive affective reactions to entrepreneurs' crowdfunding pitches: the influence of perceived product creativity and entrepreneurial passion. J. Bus. Ventur. 32, 90–106. 10.1016/j.jbusvent.2016.10.006

[B17] Decety J. (1996). The neurophysiological basis of motor imagery. Behav. Brain Res. 77, 45–52. 10.1016/0166-4328(95)00225-1 8762158

[B18] Dessart L. (2017). Social media engagement: a model of antecedents and relational outcomes. J. Mark. Manag. 33, 375–399. 10.1080/0267257X.2017.1302975

[B19] Dikker S. Wan L. Davidesco I. Kaggen L. Oostrik M. McClintock J.. (2017). Brain-to-brain synchrony tracks real-world dynamic group interactions in the classroom. Curr. Biol. 27, 1375–1380. 10.1016/j.cub.2017.04.002 28457867

[B20] Dmochowski J. P. Bezdek M. A. Abelson B. P. Johnson J. S. Schumacher E. H. Parra L. C. (2014). Audience preferences are predicted by temporal reliability of neural processing. Nat. Commun. 5, 4567. 10.1038/ncomms5567 PMC4124862 25072833

[B21] Dmochowski J. P. Sajda P. Dias J. Parra L. C. (2012). Correlated components of ongoing EEG point to emotionally laden attention-A possible marker of engagement? Front. Hum. Neurosci. 6, 112. 10.3389/fnhum.2012.00112 PMC3353265 22623915

[B22] Dulabh M. Vazquez D. Ryding D. Casson A. (2017). “Measuring consumer engagement in the brain to online interactive shopping environments,” in Augmented Reality and Virtual Reality. Progress in IS, eds T. Jung and M. tom Dieck (Cham: Springer).

[B23] Fugate D. L. (2007). Neuromarketing: a layman's look at neuroscience and its potential application to marketing practice. J. Consum. Mark. 24, 385–394. 10.1108/07363760710834807

[B24] Gabrieli J. D. E. Poldrack R. A. Desmond J. E. (1998). The role of left prefrontal cortex in language and memory. Proc. Natl. Acad. Sci. U.S.A. 95, 906–913. 10.1073/pnas.95.3.906 9448258 PMC33815

[B25] Gefen D. Straub D. W. (2004). Consumer trust in B2C e-commerce and the importance of social presence: experiments in e-products and e-services. Omega 32, 407–424. 10.1016/j.omega.2004.01.006

[B26] Hasson U. (2004). Intersubject synchronization of cortical activity during natural vision. Science 303, 1634–1640. 10.1126/science.1089506 15016991

[B27] Hasson U. Ghazanfar A. A. Galantucci B. Garrod S. Keysers C. (2012). Brain-to-brain coupling: a mechanism for creating and sharing a social world. Trends Cogn. Sci. 16, 114–121. 10.1016/j.tics.2011.12.007 22221820 PMC3269540

[B28] Hasson U. Malach R. Heeger D. J. (2010). Reliability of cortical activity during natural stimulation. Trends Cogn. Sci. 14, 40–48. 10.1016/j.tics.2009.10.011 20004608 PMC2818432

[B29] Hollebeek L. (2011). Exploring customer brand engagement: definition and themes. J. Strategic Mark. 19, 555–573. 10.1080/0965254X.2011.599493

[B30] Hou C. Wen Y. He Y. Liu X. Wang M. Zhang Z.. (2021). Public stereotypes of recycled water end uses with different human contact: evidence from event-related potential (ERP). Resour. Conservat. Recycling 168, 105464. 10.1016/j.resconrec.2021.105464

[B31] Imhof M. A. Schmälzle R. Renner B. Schupp H. T. (2017). How real-life health messages engage our brains: shared processing of effective anti-alcohol videos. Soc. Cogn. Affect. Neurosci. 12, 1188–1196. 10.1093/scan/nsx044 28402568 PMC5490672

[B32] Imhof M. A. Schmälzle R. Renner B. Schupp H. T. (2020). Strong health messages increase audience brain coupling. Neuroimage 216, 116527. 10.1016/j.neuroimage.2020.116527 31954843

[B33] Itani O. S. Kassar A. N. Loureiro S. M. C. (2019). Value get, value give: the relationships among perceived value, relationship quality, customer engagement, and value consciousness. Int. J. Hosp. Manag. 80, 78–90. 10.1016/j.ijhm.2019.01.014

[B34] KPMG (2020). A Live Streaming E-commerce Towards a Trillion Market. Available online at: https://assets.kpmg/content/dam/kpmg/cn/pdf/zh/2020/10/live-streaming-e-commerce-towards-trillion-market.pdf

[B35] Kumar V. Pansari A. (2016). Competitive advantage through engagement. J. Mark. Res. 53, 497–514. 10.1509/jmr.15.0044

[B36] Lahnakoski J. M. Glerean E. Jääskeläinen I. P. Hyönä J. Hari R. Sams M.. (2014). Synchronous brain activity across individuals underlies shared psychological perspectives. Neuroimage 100, 316–324. 10.1016/j.neuroimage.2014.06.022 24936687 PMC4153812

[B37] Landis J. R. Koch G. G. (1977). The measurement of observer agreement for categorical data. Biometrics 33, 159–174. 10.2307/2529310 843571

[B38] Madsen J. Margulis E. H. Simchy-Gross R. Parra L. C. (2019). Music synchronizes brainwaves across listeners with strong effects of repetition, familiarity and training. Sci. Rep. 9, 3576. 10.1038/s41598-019-40254-w PMC6401073 30837633

[B39] Mollen A. Wilson H. (2010). Engagement, telepresence and interactivity in online consumer experience: reconciling scholastic and managerial perspectives. J. Bus. Res. 63, 919–925. 10.1016/j.jbusres.2009.05.014

[B40] Neff J. (2007). OMD Proves the Power of Engagement. Advertising age 78. Available online at: http://www.fipp.com/News.aspx?PageIndex=2002andItemId=13735 (accessed May 17, 2010).

[B41] Park D. H. Lee J. (2008). eWOM overload and its effect on consumer behavioral intention depending on consumer involvement. Electron. Commer. Res. Appl. 7, 386–398. 10.1016/j.elerap.2007.11.004

[B42] Parra L. C. Spence C. D. Gerson A. D. Sajda P. (2005). Recipes for the linear analysis of EEG. Neuroimage 28, 326–341. 10.1016/j.neuroimage.2005.05.032 16084117

[B43] Patterson P. Yu T. de Ruyter K. (2006). “Understanding customer engagement in services,” in A*dvancing Theory, Maintaining Relevance, Proceedings of ANZMAC 2006 Conference* (Brisbane, QLD).

[B44] Poulsen A. T. Kamronn S. Dmochowski J. Parra L. C. Hansen L. K. (2017). EEG in the classroom: synchronised neural recordings during video presentation. Sci. Rep. 7, 43916. 10.1038/srep43916 PMC5339684 28266588

[B45] PwC (2020). Global Consumer Insights Survey 2020. Available online at: https://www.pwc.com/gx/en/consumer-markets/consumer-insights-survey/2020/consumer-insights-survey-2020.pdf

[B46] Sashi C. M. (2012). Customer engagement, buyer-seller relationships, and social media. Manag. Dec. 50, 253–272. 10.1108/00251741211203551

[B47] Shane S. Drover W. Clingingsmith D. Cerf M. (2020). Founder passion, neural engagement and informal investor interest in startup pitches: an fMRI study. J. Bus. Vent. 35, 105949. 10.1016/j.jbusvent.2019.105949

[B48] Smilor R. W. (1997). Entrepreneurship: reflections on a subversive activity. J. Bus. Vent. 12, 341–346. 10.1016/S0883-9026(97)00008-6

[B49] Stuss D. T. (1991). Self, awareness, and the frontal lobes: a neuropsychological perspective. Self Interdisc. Approaches 255–278. 10.1007/978-1-4684-8264-5_13

[B50] Vivek S. D. Beatty S. E. Dalela V. Morgan R. M. (2014). A generalized multidimensional scale for measuring customer engagement. J. Mark. Theory Pract. 22, 401–420. 10.2753/MTP1069-6679220404

[B51] Voyles B. (2007). Beyond loyalty: meeting the challenge of customer engagement, Economist Intelligence Unit 2007. Available online at: http://www.adobe.com/engagement/pdfs/partI.pdf (accessed January 31, 2010).

[B52] Wongkitrungrueng A. Assarut N. (2020). The role of live streaming in building consumer trust and engagement with social commerce sellers. J. Bus. Res. 117, 543–556. 10.1016/j.jbusres.2018.08.032

[B53] Young M. N. Bruton G. D. Peng M. W. Yu X. (2022). U.S. corporations are from Mars, Chinese corporations are from Venus. Bus. Horizons. 65, 505–517. 10.1016/j.bushor.2021.06.008

[B54] Yu X. Li Y. Su Z. Tao Y. Nguyen B. Xia F. (2020). Entrepreneurial bricolage and its effects on new venture growth and adaptiveness in an emerging economy. Asia Pacific J. Manag. 37, 1141–1163. 10.1007/s10490-019-09657-1

[B55] Yu X. Liu T. He L. Li Y. (2022a). Micro-foundations of strategic decision-making in family business organizations: a cognitive neuroscience perspective. Long Range Plan. 102198. 10.1016/j.lrp.2022.102198. [Epub ahead of print].

[B56] Yu X. Tao Y. Wang D. Yang M. M. (2022b). Disengaging pro-environmental values in B2B green buying decisions: evidence from a conjoint experiment. Ind. Mark. Manag. 105, 240–252. 10.1016/j.indmarman.2022.05.020

[B57] Yu X. Wang X. (2021). The effects of entrepreneurial bricolage and alternative resources on new venture capabilities: evidence from China. J. Bus. Res. 137, 527–537. 10.1016/j.jbusres.2021.08.063

[B58] Zhang M. Sun L. Qin F. Wang G. A. (2020). E-service quality on live streaming platforms: swift guanxi perspective. J. Services Mark. 35, 312–324. 10.1108/JSM-01-2020-0009

